# Nephrotoxicity after PRRT with ^177^Lu-DOTA-octreotate

**DOI:** 10.1007/s00259-016-3382-9

**Published:** 2016-05-10

**Authors:** Hendrik Bergsma, Mark W. Konijnenberg, Wouter A. van der Zwan, Boen L. R. Kam, Jaap J. M. Teunissen, Peter P. Kooij, Katya A. L. Mauff, Eric P. Krenning, Dik J. Kwekkeboom

**Affiliations:** 1 Department of Nuclear Medicine, Erasmus Medical Center, ‘s-Gravendijkwal 230, 3015 CE Rotterdam, The Netherlands; 2 Department of Biostatistics, Erasmus Medical Center, ‘s-Gravendijkwal 230, 3015 CE Rotterdam, The Netherlands

**Keywords:** PRRT, ^177^Lu-Octreotate, Kidneys, Renal function, Toxicity, Dosimetry, Nephrotoxicity

## Abstract

**Purpose:**

After peptide receptor radionuclide therapy (PRRT), renal toxicity may occur, particular in PRRT with ^90^Y-labelled somatostatin analogues. Risk factors have been identified for increased probability of developing renal toxicity after PRRT, including hypertension, diabetes and age. We investigated the renal function over time, the incidence of nephrotoxicity and associated risk factors in patients treated with PRRT with [^177^Lu-DOTA^0^,Tyr^3^]-Octreotate (^177^Lu-Octreotate). Also, radiation dose to the kidneys was evaluated and compared with the accepted dose limits in external beam radiotherapy and PRRT with ^90^Y-radiolabelled somatostatin analogues.

**Methods:**

The annual decrease in creatinine clearance (CLR) was determined in 209 Dutch patients and the incidence of grade 3 or 4 renal toxicity (according to CTCAE v4.03) was evaluated in 323 patients. Risk factors were analysed using a nonlinear mixed effects regression model. Also, radiation doses to the kidneys were calculated and their association with high annual decrease in renal function were analysed.

**Results:**

Of the 323 patients, 3 (1 %) developed (subacute) renal toxicity grade 2 (increase in serum creatinine >1.5 – 3.0 times baseline or upper limit of normal). No subacute grade 3 or 4 nephrotoxicity was observed. The estimated average baseline CLR (± SD) was 108 ± 5 ml/min and the estimated average annual decrease in CLR (± SD) was 3.4 ± 0.4 %. None of the risk factors (hypertension, diabetes, high cumulative injected activity, radiation dose to the kidneys and CTCAE grade) at baseline had a significant effect on renal function over time. The mean absorbed kidney dose in 228 patients was 20.1 ± 4.9 Gy.

**Conclusion:**

Nephrotoxicity in patients treated with ^177^Lu-octreotate was low. No (sub)acute grade 3 or 4 renal toxicity occurred and none of the patients had an annual decrease in renal function of >20 %. No risk factors for renal toxicity could be identified. Our data support the idea that the radiation dose threshold, adopted from external beam radiotherapy and PRRT with ^90^Y-labelled somatostatin analogues, does not seem valid for PRRT with ^177^Lu-octreotate.

**Electronic supplementary material:**

The online version of this article (doi:10.1007/s00259-016-3382-9) contains supplementary material, which is available to authorized users.

## Introduction

Peptide receptor radionuclide therapy (PRRT) with radiolabelled somatostatin analogues is increasingly being used in patients with neuroendocrine tumours. Frequently used somatostatin analogues are [^90^Y-DOTA^0^,Tyr^3^]-octreotide (^90^Y-DOTATOC) and [^177^Lu-DOTA^0^,Tyr^3^]-octreotate (^177^Lu-Octreotate). Although the side effects of this therapy are mild, renal toxicity has been observed, particularly in PRRT with ^90^Y-DOTATOC with an average annual decrease in creatinine clearance (CLR) of 7 % in contrast to 3 % for ^177^Lu-Octreotate [1–4]. Also several risk factors have been identified for developing renal toxicity after PRRT: poor renal function, hypertension, and diabetes at baseline [2, 5].

In the kidneys, radiolabelled somatostatin analogues are reabsorbed in the renal proximal tubules [6]. A decrease in renal uptake can be achieved by coinfusion of amino acids during PRRT [7, 8]. Despite this renoprotection, there is still a significant radiation dose to the kidneys. In the past, the threshold dose for late-stage kidney radiation damage was set at 23 Gy, which was the dose adopted from external beam radiation therapy (EBRT) [9]. According to new consensus guidelines, the limit for fractionated EBRT is set at 18 Gy that results in late radiation damage to the kidneys in 5 % of a treated population [10]. However, doses higher than 23 Gy are safely given to patients receiving PRRT with (mainly) ^90^Y-based somatostatin analogues [2, 11]. Here we present our dosimetric results and long-term follow-up of a large number of patients treated with ^177^Lu-Octreotate. We also analysed the association between known risk factors that have been indicated for PRRT with ^90^Y-based somatostatin analogues [2, 5] and change in renal function, including hypertension, diabetes, cumulative injected activity, age, previous therapies and poor renal function at baseline. In addition, radiation doses to the kidneys were calculated and analysed.

## Materials and methods

### Patients

A total of 615 patients, who were treated from January 2000 to December 2007 were studied. Inclusion criteria for the study were: patients with somatostatin positive tumours and baseline tumour uptake on [^111^In-DTPA^0^]Octreotide scintigraphy (Octreoscan®; Mallinckrodt, Petten, The Netherlands) with accumulation in the tumour at least as high as in normal liver tissue; no prior treatment with PRRT; baseline serum haemoglobin (Hb) ≥6 mmol/l; white blood cells ≥2 10^9^/l; platelets ≥75 10^9^/l; Karnofsky performance status ≥50; serum creatinine ≤150 μmol/l; and 24-h CLR ≥40 ml/min. Of the 615 patients, 323 Dutch patients were selected for this long-term evaluation, because loss to follow-up is limited in these patients.

This study was part of an ongoing prospective study in patients with neuroendocrine tumours treated with ^177^Lu-Octreotate at the Department of Nuclear Medicine, Erasmus University Medical Center Rotterdam. The hospital’s medical ethics committee approved the study. All patients gave written informed consent for participation in the study.

### Treatment

[DOTA^0^,Tyr^3^]Octreotate was obtained from BioSynthema (St. Louis, MO). ^177^LuCl_3_ was supplied by IDB-Holland (Baarle-Nassau, The Netherlands) and ^177^Lu-Octreotate was locally prepared [12].

Granisetron 3 mg or ondansetron 8 mg was injected intravenously 30 min before infusion of ^177^Lu-Octreotate. Infusion of amino acids (2.5 % arginine and 2.5 % lysine, 1 l) was started 30 min before administration of the radiopharmaceutical and lasted for 4 h. The radiopharmaceutical was coadministered for 30 min using a second pump system. The interval between treatments was 6 – 16 weeks. The intended cumulative activity was 29.6 GBq (800 mCi). Median cumulative activity was 29.6 GBq (range 7.4 – 29.6 GBq) and the median number of therapy cycles was four (range one to eight). However, the total administered activity was lowered if the calculated kidney dose was higher than 23 Gy. Other reasons for dose reduction or cessation of further therapy were recurrent grade 3 or 4 haematological toxicity and persistently low blood cell counts.

### Toxicity, risk factor assessment and follow-up

Haematology, liver and renal function tests were performed during the 6 weeks before the first therapy, 4 and 6 weeks after each therapy, and at follow-up visits. Acute and long-term renal toxicity assessment was done according to Common Terminology Criteria for Adverse Events (CTCAE v4.0) [13].

Hypertension was defined as the use of antihypertensive drugs (thiazide diuretics, beta blockers, ACE inhibitors, angiotensin II receptor antagonists and calcium channel blockers). Diabetes mellitus was defined as an HbA1c of ≥6.0 % and/or the use of antidiabetic medication (insulin and insulin sensitizers). CLR in millilitres per minute was used as an estimate of glomerular filtration rate (GFR). Four serum-based methods were used to determine baseline 24-h urine CLR, and the results compared (see Supplementary material). The Cockcroft-Gault (CG) formula had the highest Spearman’s rank correlation coefficient. Therefore, changes in renal function during follow-up were assessed in terms of CLR determined using the CG formula:

$$ CLR\left( ml/ min\right)=\frac{140- age\left[y\right]\cdot weight(kg)}{s- creatinine\left(\mu mol/L\right)}\cdot \left[0.85\kern.2em  if\kern.2em f\kern-.2em  emale\right] $$

### Statistical analysis

SPSS (SPSS 19; IBM, Armonk, NY) and R (R 3.2.2; R Foundation for Statistical Computing, Vienna, Austria) software was used for statistical analysis. The Shapiro-Wilk test was used to assess the normality of the response. The primary outcome was CLR predicted by a nonlinear mixed effects regression model with independent factors (hypertension, diabetes, cumulative injected activity, radiation dose to the kidneys, age and CTCAE at baseline). Various functional model forms (linear, nonlinear, polynomial and spline) were fitted to the CLR data (see Supplementary material). The nonlinear model with the monoexponential function performed best

where $\widehat{b_0}$ is the estimated average CLR at time 0 when all other covariates are zero, and $\widehat{b_1}$ is the estimated average change in CLR in percent/time. Time is expressed in weeks and factor_1_ and factor_2_ are constants, given specific values of the covariates included in the mixed model. The combined term $\widehat{\Big(b_0}\bullet {factor}_1\Big)$ represents the estimated average CLR at time = 0 for a specific covariate pattern, whereas $\widehat{\Big(b_1}\bullet {factor}_2\Big)$ is the estimated average percentage decrease/increase in CLR per week. Random effects were included on both the intercept and slope parameters, and a diagonal covariance matrix was assumed.

### Dosimetry

Uptake of radioactivity in the kidneys was determined by planar imaging at 1, 3 – 4 and 7 days after administration of ^177^Lu-Octreotate. Extensive information regarding the dosimetric method is provide in an earlier paper [12]. Dosimetry values were computed with *S*-factors for ^177^Lu derived from the Radiation Dose Assessment Resource (RADAR) website [14, 15]. The general scheme for calculating radiation dosimetry with radionuclides has been defined by the MIRD scheme dosimetry formula [16]:

$$ \begin{array}{l}D\left({r}_t\right)={\displaystyle \sum_S{\displaystyle \int {A}_S(t)dt\cdot S\left({r}_t\leftarrow {r}_S\right)}}\hfill \\ {}={\displaystyle \sum_S}{\tilde{A}}_S\cdot S\left({r}_t\leftarrow {r}_S\right)=\gg {D}_{kidneys}\approx {\tilde{A}}_{kidneys}\cdot S\left( kidneys\leftarrow kidneys\right)\hfill \end{array} $$

The dose to the target organ (*D*_kidneys_) is calculated as the product of the number of decays in a source organ (*Ã*_s_) and the *S*-value, which expresses the dose rate per radioactivity for a source (*r*_s_) to target (*r*_t_) combination. With moderately weak β-particle-emitting radionuclides such as ^177^Lu, only the self-dose needs to be considered (*r*_s_ =* r*_t_). The radioactivity uptake and clearance kinetics of ^177^Lu-Octreotate *A*_s_(t) in the kidneys is needed for calculation of the radiation dose to the kidneys, together with the *S*-value for the kidney self-dose. The ^177^Lu *S*-values were taken from the RADAR website [15]: for adult male kidneys (with a mass of 299 g) the *S*-value is 0.289 mGy/MBq.h and for a adult female kidneys (with a mass of 275 g) the *S*-value is 0.314 mGy/MBq.h. In a subgroup of patients, the kidney volume was determined based on baseline CT images, since complete kidney imaging was not always available. A correction factor and adjusted dose were calculated in these patients, using OsiriX 5.9 (Pixmeo Sàrl, Bernex, Switzerland). A polygonal region of interest was (semi)automatically drawn on each CT slice and the slices were summed for calculation of the total kidney volume.

## Results

**Fig. 1:**
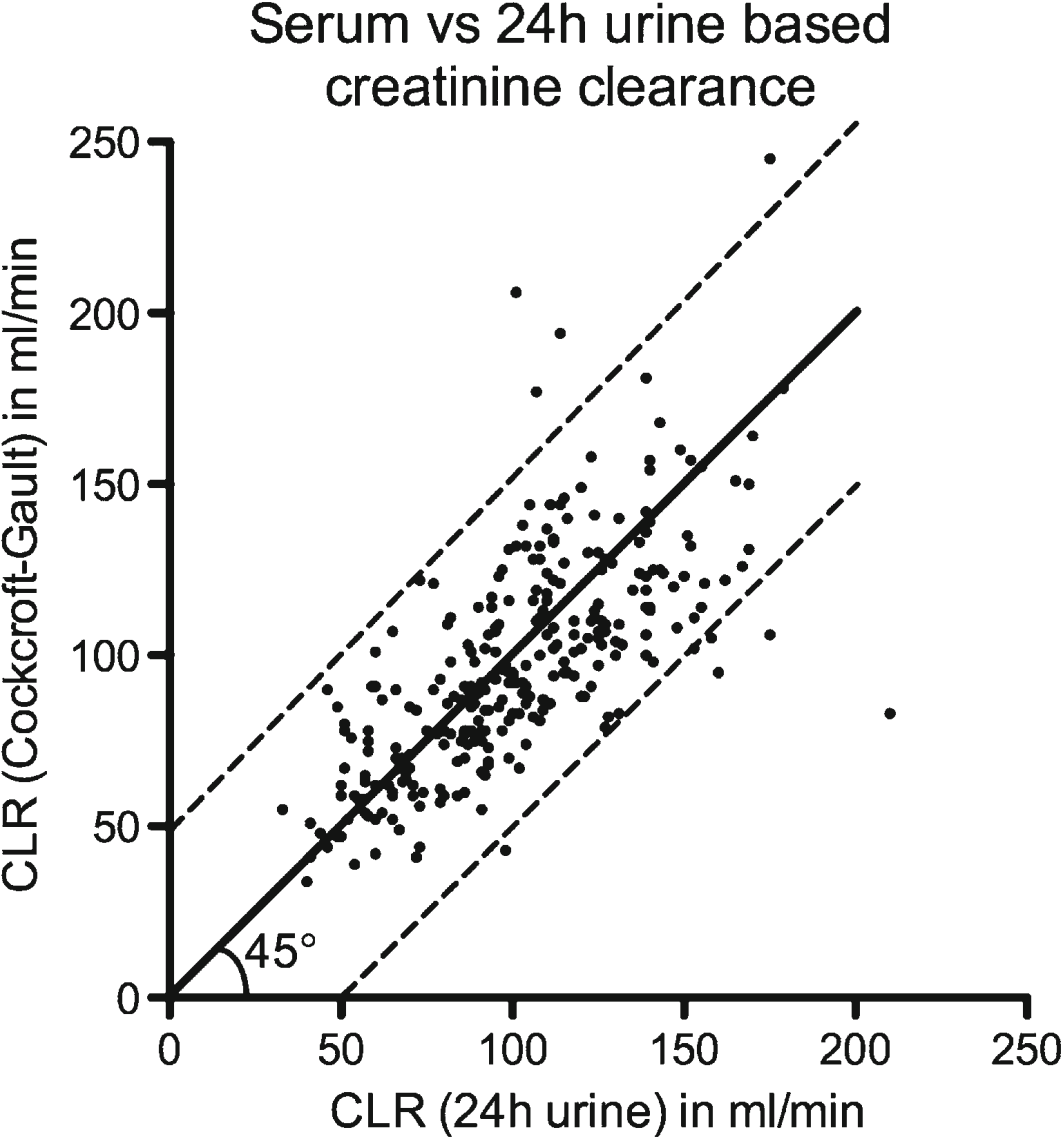
Baseline 24-h urine creatinine clearance (CLR) versus serum-based CLR according to the Cockcroft-Gault formula in 281 of 323 patients. The *solid line* is the linear regression line with a slope of 1 with 95 % confidence intervals (*dotted lines*)

In 554 patients the inclusion criteria were met. In-depth evaluation was done in 322 Dutch patients (excluding one patient with no baseline CLR). Patient characteristics are summarized in Table 1. A Spearman’s correlation coefficient of 0.76 was found between baseline 24-h urine and serum-based CLR (Fig. 1).

**Table 1:** Baseline characteristics of 323 Dutch patients

Characteristic	no.
Gender	
Male	158 (49 %)
Female	165 (51 %)
Age (years)	
≥70	63 (20 %)
<70	260 (80 %)
Karnofsky performance status	
≤70	46 (14 %)
>70	277 (86 %)
Diabetes	
Yes	104 (32 %)
No	219 (68 %)
Hypertension	
Yes	77 (24 %)
No	246 (76 %)
Solitary kidney	
Yes	10 (3 %)
No	313 (97 %)
Previous therapy	
Radiotherapy (external)	
Yes	32 (10 %)
No	291 (90 %)
Chemotherapy	39 (12 %)
Cisplatin	5 (13 %)
Other	34 (87 %)
Tumour type	
Neuroendocrine tumour	281 (87 %)
Other	42 (13 %)
Dosimetry	
Dosimetric data available	228 (71 %)
Limit 23 Gy to the kidneys	
Yes	55 (24 %)
No	173 (76 %)
Volume of kidneys available	
Yes	119 (52 %)
No	109 (48 %)
Cumulative activity (GBq)	
Up to 22.2	106 (33 %)
Up to 29.6	217 (66 %)
Kidneys	
Baseline creatinine clearance	
<60 ml/min/1.73 m^2^	37 (11 %)
≥60 ml/min/1.73 m^2^	286 (89 %)
Baseline Cockcroft-Gault creatinine clearance (ml/min), median (range)	95 (34 – 245)
Follow-up (months), median (range)	25 (0 – 142)

### Kidney toxicity

Of the 323 patients, 14 (4 %) had a (sub)acute toxicity grade 1 (creatinine increase >26.5 µmol/l). Three patients (1 %) developed (subacute) toxicity grade 2 (creatinine increase >1.5 – 3.0 × baseline or upper limit of normal). These were judged not related to therapy: one patient had received prolonged antibiotics due to an infection and developed temporary renal insufficiency, one patient was dehydrated because of diarrhoea, and one patient showed progression of disease with hypoalbuminaemia and forward heart failure resulting in death 2 weeks after the first treatment. No grade 3 or 4 (sub)acute nephrotoxicity was observed.

**Fig. 2:**
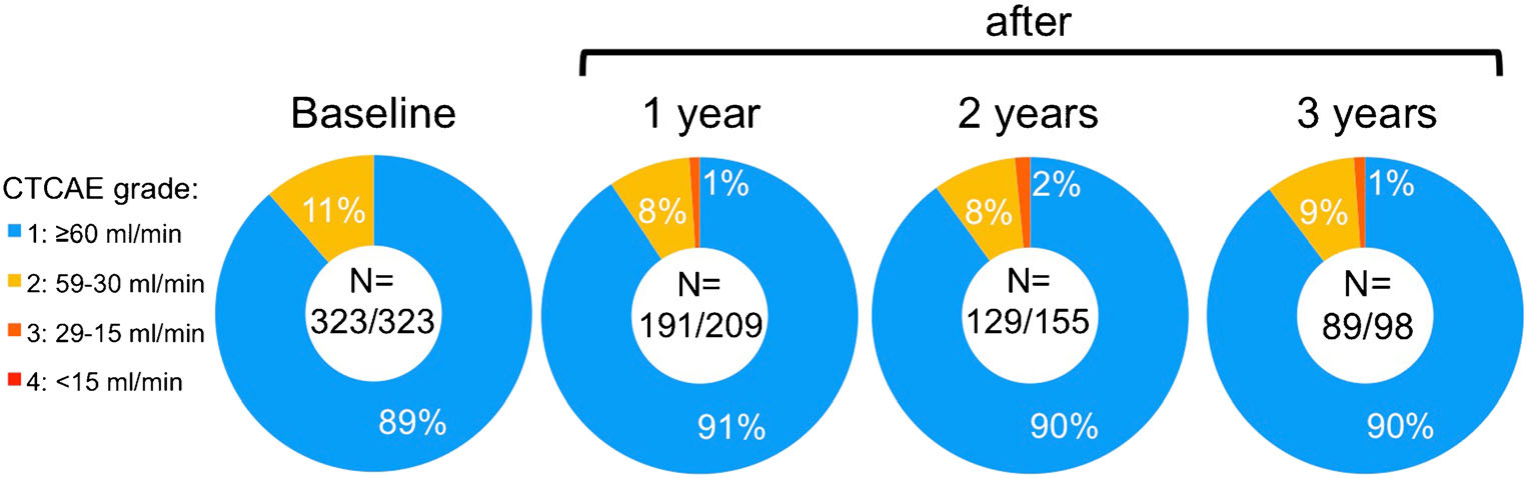
Distribution of creatinine clearance in 323 patients according to Common Terminology Criteria for Adverse Events (CTCAE) classification at baseline, and at 1, 2 and 3 years after inclusion. Number (N) of patients with serum creatinine available / total number of patients in follow-up. No CTCAE grade 4 was observed

Follow-up data for 1, 2 and 3 years after the last therapy were available in 209, 155 and 98 patients, respectively. Grade 3 kidney toxicity was observed in 5 out of the 323 patients during this follow-up. Toxicity was not related to PRRT since all five patients had a baseline CLR of <60 ml/min (i.e. grade 2), making them more prone to more severe renal function impairment. However, the annual decrease in CLR was <12 %. The distribution of CLR at baseline and during follow-up (1, 2 and 3 years after inclusion) is shown in Fig. 2. Reasons for loss to follow-up after 1, 2 and 3 years are summarized in Table 2. One patient was lost to follow-up after 2 years, due to kidney failure resulting in dialysis based on preexisting kidney disease.

**Table 2:** Cumulative numbers of 323 Dutch patients lost to follow-up 1, 2 and 3 years after the last PRRT

Reason lost to follow-up	After 1 year	After 2 years	After 3 years
Progressive disease	43	47	56
Death	8	9	12
Follow-up elsewhere (patient request)	18	23	32
Complications (e.g. bleeding, infection, ileus, dyspnoea)	11	13	19
Bone marrow suppression	4	7	9
Liver failure	2	2	2
Other therapy	23	28	40
Octreoscan-negative lesions during follow-up	2	2	3
Retreatment with ^177^Lu-DOTATATE	3	37	51
Kidney failure (see text)	0	0	1
Total number of patients	114	168	225

### Long-term change in renal function

**Fig. 3:**
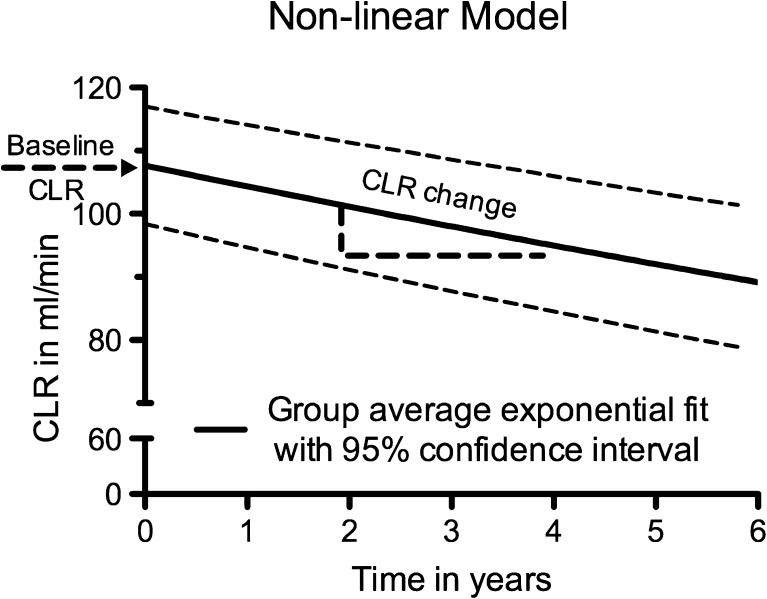
Nonlinear model of creatinine clearance (CLR) over time based on 208 patients. *Solid line* is the exponential function with 95 % confidence interval (*dashed lines*). The estimated average baseline CLR (± SD) is 108 ± 5 ml/min and the estimated average annual change in CLR (± SD) is 3.4 ± 0.4 %

**Fig. 4:**
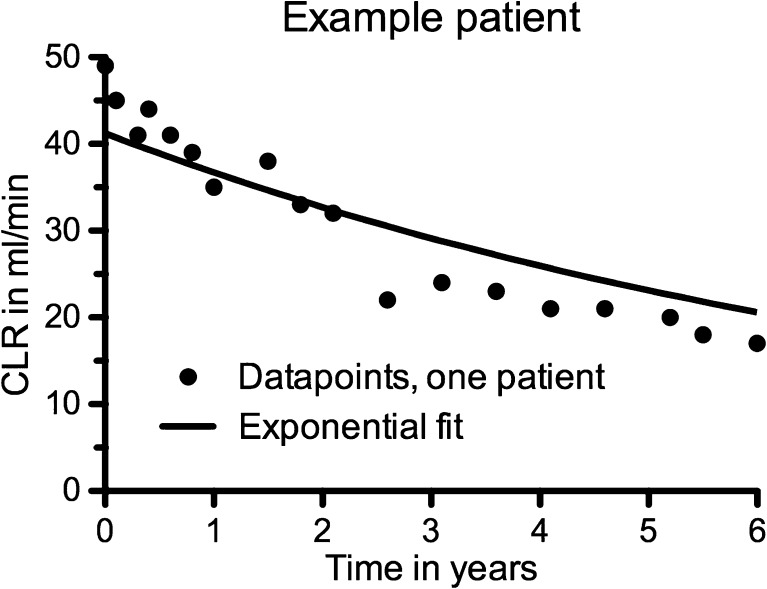
Time-course of creatinine clearance (CLR) and fitted monoexponential decay (*solid line*) in a 71-year-old patient with a neuroendocrine tumour, hypertension and diabetes, who received 4 × 7.4 GBq ^177^Lu-Octreotate. The estimated decrease in CLR is 11.4 % per year

**Fig. 5:**
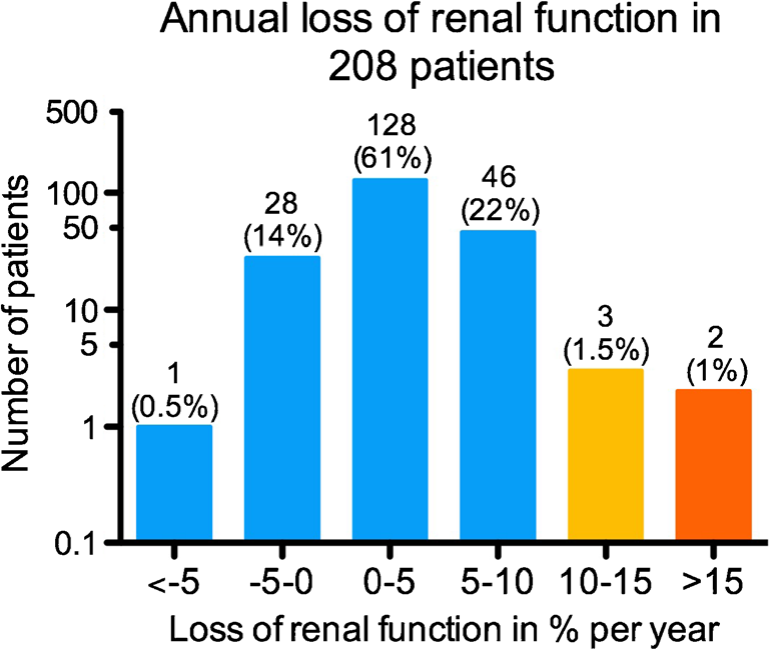
Distribution of the change in creatinine clearance per year in 208 patients with long-term follow-up. Note the log scale on the *y*-axis. Coloured bars represent annual loss of renal function < 10% (blue), 10-15% (yellow) and >15% (orange)

Follow-up of more than 1 year was available in 209 of the 323 patients. One patient with an incomplete set of risk factors was excluded; thus the analysis included 208 patients. The estimated average annual decrease in CLR (± SD) was 3.4 ± 0.4 %, and the estimated average baseline CLR ($\widehat{b_0}$) was 108 ± 5 ml/min (Fig. 3). The time course of CLR and the fitted nonlinear model in an example patient are shown in Fig. 4. In 203 out of 208 patients, the annual decrease in renal function was <10 %. Five patients had an annual decrease in CLR of ≥10 %, and two patients had an annual decrease of ≥15 % (Fig. 5). In 29 (14 %) of 208 patients, a positive annual change in CLR (improvement in renal function) was observed. No patient showed an annual decrease in renal function of ≥20 %.

### Risk factor assessment

Age and baseline CTCAE had significant effects on the baseline CLR (both *p* < 0.0001). With all other factors held constant, the estimated average baseline CLR showed a significant decrease in patients older than 70 years or with baseline CTCAE grade 2. None of the risk factors considered for inclusion in the model (hypertension, diabetes, age >70 years, cumulative injected activity >22.2 GBq, high radiation dose to the kidneys and CTCAE grade at baseline) had a significant effect on the estimated rate of change in CLR over time, and were thus not included in the final model.

### Dosimetry

**Fig. 6:**
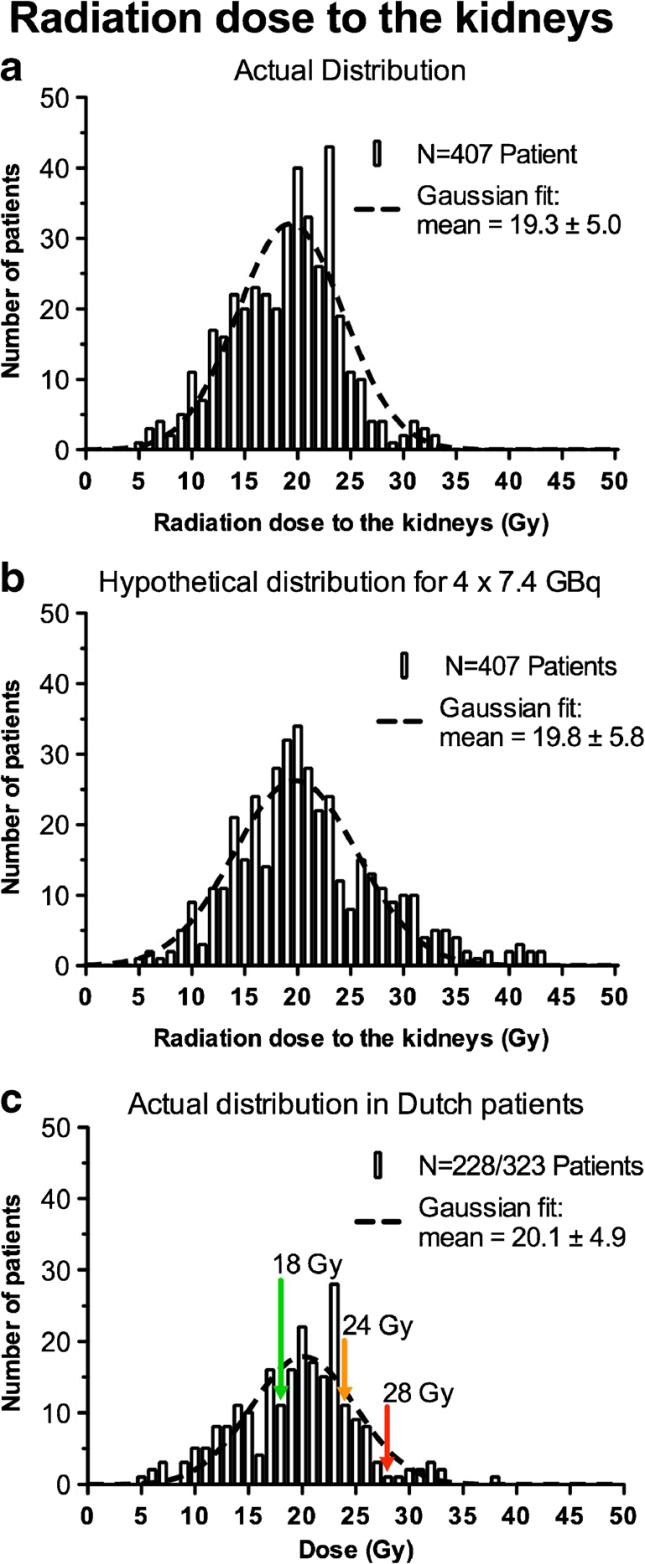
Distribution in 1-Gy increments of the radiation dose to the kidneys for 407 patients and in 228 patients with quantifiable kidney uptake: **a** actual distribution in 407 patients; **b** hypothetical distribution for 4 × 7.4 GBq of ^177^Lu-Octreotate; **c** actual distribution in 228 Dutch patients. Gaussian fits (*dashed lines*) are overlain on the histograms. The *green arrow* indicates the kidney threshold dose (18 Gy) according to current EBRT guidelines [10]. The *orange arrow* (24 Gy) and *red arrow* (28 Gy) correspond to the PRRT dose limits for kidney damage according to Wessels et al. [11] and Bodei et al. [2], respectively, for therapies given in four cycles

Dosimetric data for kidney dose calculations was available in 407 of the 554 on-protocol patients. In 147 patients no radiation dose to the kidneys could be calculated due to incomplete dosimetric data and/or over-projection of tumour nodules on planar images of the kidney region of interest. Clearance of radioactivity from the kidneys proceeded with a median effective half-life of 58 h (range: 27 – 135 h) in 407 patients. The mean radiation dose to the kidneys was 19.3 ± 5.0 Gy (Fig. 6a). The mean kidney absorbed dose for a hypothetical dose distribution of 4 × 7.4 GBq of ^177^Lu-Octreotate was 19.8 ± 5.8 Gy (Fig. 6b). The mean calculated radiation dose to the kidneys in 228 of the 323 Dutch patients in whom it could be calculated was 20.1 ± 4.9 Gy (Fig. 6c), and 11 (5 %) of these 228 patients had a calculated kidney absorbed dose of more than 28 Gy. The total administered injected activity was reduced in 55 of the 228 patients because the calculated kidney dose was more than 23 Gy. The average measured kidney volume (with corresponding mass) in 119 (49 %) of the 228 patients in whom it could be measured was a factor of 0.95 (range 0.49 – 1.71) less than the fixed phantom-based kidney mass.

## Discussion

After PRRT with ^177^Lu-Octreotate, the average annual decrease in CLR was 3 % and no patient showed a decrease of more than 20 %, which is in line with the results of other studies with ^177^Lu-Octreotate [2, 5, 17, 18]. Of the patients treated with ^177^Lu-Octreotate, 14 % showed an annual improvement in CLR. Tumour response and improvement in clinical condition could explain the increase in CLR in these patients, since a rapid weight gain with stable serum creatinine values results in a higher CLR. Therefore, we suspect that the improvement in CLR did not reflect a true improvement in renal function.

In practice, an annual decrease of 3 % means a CLR of 91 ml/min after 3 years in a patient with normal renal function at baseline (Table 3). Five of our patients had an annual decrease in renal function of more than 10 %, translating to a CLR after 3 years of 74 ml/min in an individual with normal renal function at the start. Since the overall survival following PRRT with ^177^Lu-octreotate is 3 to 4 years [19], it is unlikely that the kidneys are the long-term limiting factor. A decrease in renal function to CTCAE grade 2 or higher occurs after 7 years in a patient with normal renal function at the start and an annual decrease of 10 % (Table 3). In our study, only 1 % of the patients developed therapy-unrelated severe (grade 3) renal toxicity after 1 year. Furthermore, the CTCAE distribution of CLR over time did not change, confirming the low nephrotoxicity of PRRT with ^177^Lu-Octreotate.

**Table 3:** Creatinine clearance in hypothetical patients with a baseline renal function of 100 ml/min and annual decreases of 3 %, 10 % and 20 %

Year	Creatinine clearance (ml/min)		
	3 % annual decrease	10 % annual decrease	20 % annual decrease
0	100	100	100
3	91	74	55^a^
5	86	61	37^a^
7	81	50^a^	25^a^

^a^Creatinine clearance of CTCAE grade 2 or higher

Several risk factors for kidney toxicity after PRRT with mainly ^90^Y-labelled somatostatin analogues have been identified, including age (>60 years), diabetes, hypertension, previous chemotherapy and poor baseline renal function [1, 2, 4]. In a recent study [5], renal function was analysed in 807 patients treated with ^177^Lu-Octreotate and/or ^90^Y-Octreotide. Hypertension was identified as a main risk factor for (persistent) nephrotoxicity. However, nephrotoxicity was defined as a categorical outcome according to CTCAE, neglecting the change in renal function over time. This can lead to a simplified representation of kidney toxicity after PRRT and false identification of risk factors. Valkema et al. analysed renal function in 37 patients treated with ^177^Lu-Octreotate by taking the subject-specific annual decrease in CLR extracted from fitted monoexponential curves [1]. Hypertension was also found to be a possible factor contributing to the rate of decrease in CLR after PRRT. Although the authors did take into account the change in renal function over time, they were unable to determine the impact of covariates on the change over time but only on summary measurements obtained from individually fitted curves.

 Given the repeated measurement structure of the data and the need to assess the effect of risk factors on both baseline CLR and change in CLR over time, a more advanced approach is required. We therefore used a mixed effects regression model. Mixed effects models are the standard modelling framework for the analysis of longitudinal data. These models explicitly account for differences in correlation structure of the data within/between patients and deal well with unbalanced data (varying times of measurement in each subject and unequal numbers of follow-up measurements among subjects).

In our present analysis, age >70 years and baseline CTCAE grade influenced the nonlinear model. However, the two risk factors only changed the estimated average baseline CLR component in our model, meaning that patients older than 70 years and/or patients with baseline CTCAE grade 2 had a lower estimated average CLR at baseline. None of the evaluated risk factors modified the percentage CLR change component significantly, implying that the percentage change in CLR over time in patients with risk factors was not different from that in patients without risk factors.

An explanation for our results could be that the frequency of nephrotoxicity after PRRT with ^177^Lu-Octreotate is low and a higher number of patients would be required to show statistical significance of the risk factors. For the same reason, we were not able to compare patients with/without a solitary kidney and with/without alkylating chemotherapy (e.g. cisplatin). The relatively low numbers of patients resulted in low statistical power for testing these risk factors.

GFR measurement with inulin is the gold standard for measuring renal function, but practical implementation is difficult [20]. Other radionuclide-based filtration markers such as ^99m^Tc-MAG3 (mercaptoacetyltriglycine) are used for accurate assessment of renal function in clinical practice [21]. However, these methods are expensive and time-consuming in the follow-up of large patient groups. We used CLR as an indirect marker for estimating GFR since serum creatinine is widely available. Also most of our patients had normal baseline renal function, making CLR a reasonable estimator for GFR. However, different formulas for calculation of renal function are available: the (body surface area-corrected) CG formula and the (abbreviated) modification of diet in renal disease (MDRD) equations. The CG formula estimates CLR [22], whereas the MDRD equations estimate GFR [23]. All formulas have different performance in various subgroups of patients depending on age, sex, weight and range of renal function [24]. Therefore, we calculated Spearman’s rank correlation coefficients for different equations versus 24-h urine CLR in our patient group. The CG formula had the best correlation (Fig. 1). Our results are in line with those of other studies indicating that CG is more precise than MDRD [24], meaning that individual changes in renal function over time are more reliable.

Radiation toxicity dose effect models used in PRRT are predominantly based on the experience and knowledge obtained from EBRT. In the past, the threshold dose for late-stage kidney radiation damage for EBRT was set at 23 Gy [9]. However, the tolerable dose in current guidelines for radiotherapy-associated kidney injury are lower at 18 Gy [10]. Kidney radiation doses of 18 Gy given in a fractionation scheme of 2 Gy per fraction are considered to result in a 5 % probability of developing radiation nephropathy during the 5 years after EBRT.

For PRRT with ^90^Y-DOTATOC, a correlation was found between the kidney absorbed dose and chronic kidney toxicity. The dose at which 5 % of patients will show kidney toxicity has been estimated at 24 Gy for ^90^Y-DOTATOC [11]. Another study has confirmed this dose limit in 22 of 50 patients treated with ^90^Y-DOTANOC [25]. The absorbed dose limit after ^90^Y irradiation and that for fractionated EBRT can be compared using the concept of the biologically effective dose (BED). BED is a measure of the true biological dose delivered at a particular dose rate and fractionation pattern, tissue-specific for a relevant biological end-point (in this case late-stage renal disease). It takes the protracted nature of the absorbed dose delivery by radionuclides into account by adjusting the kidney’s radiation sensitivity for the repair of sublethal radiation damage during the absorbed dose build-up according to the linear quadratic (LQ) model. The BED concept is thought to explain the 6-Gy higher dose limit than the 18 Gy accepted for EBRT [10]. Bodei et al. proposed a BED limit of 40 Gy to the kidneys in patients without risk factors and a BED of 28 Gy in patients with risk factors, corresponding to absorbed doses of 28 and 24 Gy, respectively (both given in four fractions) [2]. A summary of previously reported kidney dosimetry findings in studies using ^177^Lu-Octreotate is provided in Table 4.

**Table 4:** Reported data on kidney dosimetry for PRRT with ^177^Lu-Octreotate

Reference	Method	No. of patients	Administered activity (GBq)	Amino acids	Dose to kidneys	
					Per activity administered (Gy/GBq)	For 4 × 7.4 GBq (Gy)
[12]	Planar	5	1.85	Lys/Arg	0.9 ± 0.2	26.6 ± 5.3
[2]	Planar	5	3.7 – 5.18	Not reported	0.9 ± 0.5	26.6 ± 13.3
[32]	Planar	69	3 – 7	Lys/Arg	0.9 ± 0.3	26.6 ± 8.0
[33]	SPECT/CT	24	7.4	Vamin 14	0.7 ± 0.3	20.7 ± 6.2
[34]	SPECT/CT	16	7.4	Vamin 14	0.9 ± 0.3	26.6 ± 8.0
[35]	Planar	26	8	Not reported	0.9 ± 0.4	26.6 ± 10.6
[36]	SPECT/CT	33	7.8	Synthamin	0.3 (0.1 – 0.5)	9.2 (4.1 – 13.6)
[18]	Planar	12	5.18 – 7.4	Lys/Arg	0.8 ± 0.4	23.7 ± 9.5
[37]	SPECT/CT	200	7.4	Vamin 14	1.2 ± 0.6	36.3 ± 16.0
[28]	Planar	51	3.5 – 8.2	Lys/Arg	0.8 ± 0.4	23.7 ± 9.5
This study	Planar	407	7.4	Lys/Arg	0.7 ± 0.2	19.8 ± 5.8

Values are means ± SD, or median (range)

*Lys/Arg* Lysine 2.5 % and arginine 2.5 %, *Lys* Lysine 2.5 %

In this study, we did not find a significant difference in the effect of kidney dose on renal function. Most patients received a kidney dose of less than 28 Gy (Fig. 6c). However, in a small number of patients the kidney dose exceeded this limit. In our long-term follow-up group, 11 patients received a kidney dose that exceeded 28 Gy. None of these patients developed grade 3 or 4 nephrotoxicity and/or had an annual decrease in CLR of more than 10 %. Therefore, the 28 Gy dose limit seems to be a conservative value for PRRT with ^177^Lu-Octreotate. Another argument for a higher dose limit is that ^177^Lu has shorter range β-particles than ^90^Y. This results in less damage to nearby nontarget tissue and (theoretically) in fewer cases of nephrotoxicity at a fixed kidney dose [26–28].

In PRRT studies, the LQ model concept of BED was first introduced with dosimetric data from 18 patients who received ^90^Y-DOTATOC [29]. A stronger correlation was observed with the decrease in CLR when applying this model, compared with using absorbed renal dose alone. Also, a comparison between the relatively high dose-rate of EBRT and low dose-rate irradiation of radionuclide therapy is possible using BED. The LQ model can be used to analyse the effects of dose rate, number of therapy cycles in EBRT/PRRT and the type of radionuclide in PRRT. The LQ model-based BED for PRRT has been adopted by the Committee on Medical Internal Radiation Dose (MIRD) for late kidney damage [11]. In PRRT little scientific evidence is available for the choice of α/β ratio and repair T_1/2_, which represents the damage and repair half-life in the BED model, however the two variables have an important effect on the dosimetric outcome [30, 31]. Also, since no severe renal toxicity was observed in the present study with an intended dose scheme of 4 × 7.4 GBq, reporting the BED did not seem appropriate. Moreover, in the present study the factor relating absorbed dose and BED was low: median 1.09 (range 1.02 – 1.21).

Our results demonstrate that the kidneys are not the dose-limiting organ in patients treated with ^177^Lu-Octreotate. Therefore, in clinical practice, kidney dosimetry does not currently have a prominent place in PRRT with 4 × 7.4 GBq ^177^Lu-Octreotate. However, (serum-based) assessment of renal function during and after PRRT is mandatory since renal toxicity unrelated to PRRT could occur. Also measurement of renal function at baseline is required since low GFR is a risk factor for development of (sub)acute haematotoxicity after PRRT.

### Conclusion

The number of patients with nephrotoxicity after PRRT with ^177^Lu-Octreotate is low. No patient showed (sub)acute grade 3 or 4 nephrotoxicity or an annual decrease in renal function of >20 %. No risk factors (e.g. hypertension, diabetes) leading to an additional annual decrease in renal function could be identified. Our study showed that the maximum radiation dose to the kidney adopted from EBRT and PRRT with ^90^Y-labelled analogues does not seem to apply to PRRT with ^177^Lu-Octreotate.

## Electronic supplementary material

Below is the link to the electronic supplementary material.

ESM 1 (DOCX 914 kb)
