# Supplementary material for: Nephrotoxicity after PRRT with 177Lu-DOTA-octreotate
Source: Eur J Nucl Med Mol Imaging. 2016 May 10;43:1802–11. doi: 10.1007/s00259-016-3382-9 (PMC4969358; doi:10.1007/s00259-016-3382-9)
Supplement: Supplementary file 1 — (DOCX 914 kb) [file 259_2016_3382_MOESM1_ESM.docx]

**Supplemental Data**

**Nephrotoxicity after PRRT with ^177^Lu-DOTA-Octreotate**

Hendrik Bergsma^1*^, Mark Konijnenberg^1*^, Wouter A. van der Zwan^1^, Boen L.R. Kam^1^, Jaap J.M. Teunissen^1^, Peter P. Kooij^1^, Katya A.L. Mauff ^2^, Eric P. Krenning^1^ and Dik J. Kwekkeboom^1^;

Departments of ^1^ Nuclear Medicine and ^2^ Biostatistics, Erasmus Medical Center, ‘s-Gravendijkwal 230, 3015 CE Rotterdam, The Netherlands

For correspondence or reprints contact: Hendrik Bergsma MD, Department of Nuclear Medicine, ErasmusMC, Department of Nuclear Medicine, Erasmus University Medical Center Rotterdam, 's Gravendijkwal 230, 3015 CE Rotterdam - The Netherlands.

E-mail: bergsmahb@gmail.com, Telephone: +31 10 704 0132, Fax: +31 10 703 4647

* Contributed equally with Mark Konijnenberg, PhD, Department of Nuclear Medicine, Erasmus MC, Rotterdam, The Netherlands.

**Methods**

*Follow-up and data analyses*

Creatinine clearance (CLR) was used as an estimate of the glomerular filtration rate (GFR). Baseline 24hr urine creatinine was collected and compared with different serum based creatinine clearance methods. We estimated the serum CLR with four formulas (see below) in comparison to 24-h urine CLR at baseline.

24hr urine collection:

$$GFR=C_{x}=\frac{U_{creatinine}}{s-creat(\mu mol/L)}$$

Cockcroft-Gault (C-G):

$$c-CLR=\frac{(140-age[y]\cdot weight(kg)}{s-creat\left( \mu mol/L \right)}\cdot[0.85 if female]$$

Cockcroft-Gault (C-G) correct for body surface area (BSA):

$$c-CLR=\frac{(140-age[y]\cdot weight(kg)\cdot[0.85 if female]}{s-creat\left( \frac{\mu mol}{L} \right)}\cdot\frac{1.73}{BSA}$$

Body surface area (BSA)according to Du Bois and Du Bois:

$$BSA=({body-weight}^{0.425}\left[ in kg \right]\cdot{height}^{0.725}\left[ in cm \right])\cdot0.007184$$

Modification of Diet in Renal Disease (MDRD):

$$GFR=186\cdot{(s-creat\cdot0.0113)}^{-1.154}\cdot{Age}^{-0.203}\cdot(0.742 if female)\cdot(1.21 if black)$$

Extended Modification of Diet in Renal Disease (extMDRD):

$$GFR=170\cdot{(s-creat\cdot0.0113)}^{-0.999}\cdot{Age}^{-0.176}\cdot{(BUN\cdot2.80)}^{-0.170}\cdot({Alb\cdot0.1)}^{0.318}\cdot(0.762 if female)\cdot(1.18 if black)$$

where BUN is the blood urea nitrogen in mmol/l and Alb is the albumin in g/l.

The difference between estimated and measured CLR from 24h-urine was the smallest in the C-G formula (figure 1). Also the spearman rank correlation coefficient was the best in the latter (figure 2).

*Dosimetry*

All patients underwent planar imaging to determine kidney dosimetry. Dosimetry was performed minimally after the first treatment to determine if the 4th treatment with 7.4 GBq of 177Lu-DOTA0-Tyr3-Octreotate would result in a kidney radiation dose that would not exceed the 23 Gy threshold limit. 407 of the 554 on-protocol patients had quantifiable kidney uptake, which enabled kidney dosimetry. The lack of accurate dosimetry data for the remaining patients is largely due to overlapping radioactivity from tumours in the abdominal region obscuring organ delineation.

The ^177^Lu S-values were taken from the Radiation Dose Assessment Resource (RADAR) website [1]: for a male adult (with 300g kidneys) the S-value is 0.289 mGy/MBq.h and for a female adult (with 275 g kidneys) the S-value is 0.314 mGy/MBq.h. These can be adjusted with the actual patient’s kidney volume, derived from CT imaging [2]. A correction factor and adjusted dose was calculated in patients with CT scan available before the first treatment, using OsiriX 5.9 (Pixmeo SARL, Bernex – Swiss). A polygon region of interest (ROI) was (semi)automatic drawn on each CT slice and each slice was summed up for calculation of the total kidney volume.

**Imaging**

Planar spot images of the upper abdomen, chest, and the head and neck region were obtained with a double head camera (Picker Prism 2000) at regular intervals. For the first administration in Group-1, the imaging time points were 4 h, and 1, 3, 10 and 17 days after administration. After the second administration in Group-1 (with amino acid co-infusion) and in all other groups, the imaging time points were 1, 3 or 4 and 7 or 10 days after administration. Counts from both gamma-peaks (208 and 113 keV) were collected in separate windows (width 20%). Acquisition time was 15 min/view at maximum. A standard with a known aliquot of the infused radioactivity was also counted for dosimetry calibration.

**Regions of Interest**

For biodistribution studies the percentage of infused radioactivity (%IA) accumulated in each organ was measured by manually contouring and selecting the regions of interest (ROIs) for the whole body, bone marrow, kidney, liver, spleen, total abdomen, thyroid, gonads, pituitary, adrenals and pancreas. The geometric means of the regions were computed and these values were used to calculate the %IA based on a comparison to an aliquot of the administered dose [3, 4].

**Dosimetry Calculations**

Dosimetry values were computed with a MIRDOSE software package, version 3.0, and using S-factors for ^177^Lu [5]. The general scheme for calculating radiation dosimetry with radionuclides has been defined by the MIRD scheme dosimetry formula [6].

The dose to the target organ (D; kidney or bone marrow) is calculated by the product of the number of decays in a source organ (*Ã_s_*) and the S-value, which expresses the dose rate per radioactivity for a source (r_s_) to target (r_t_) combination. With moderately weak beta-particle emitting radionuclides like ^177^Lu, only the self-dose needs to be considered (*r_s_=r_t_*). The radioactivity uptake and clearance kinetics of ^177^Lu-DOTA^0^-Tyr^3^-Octreotate *A_s_(t)* in the kidneys is needed for calculation of the radiation dose to the kidneys, together with the S-value for the kidney self-dose. The ^177^Lu S-values were taken from the Radiation Dose Assessment Resource (RADAR) website [1]: for a male adult (with 300g kidneys) the S-value is 0.289 mGy/MBq.h and for a female adult (with 275 g kidneys) the S-value is 0.314 mGy/MBq.h. These can be adjusted with the actual patient’s kidney volume, derived from CT imaging [2], this was not done in a select number of patients.

**Results**

*Statistical analysis*

Baseline 24hr urine CLR was compared with 4 serum based CLR methods (Appendix A). The Cockcroft-Gault (CG) formula had the highest Spearman rank correlation coefficient (see figure 1 & 2).

*Dosimetry*

In 228 out of 323 Dutch patients the radiation dose to the kidneys (and SD) was 20.1 ± 4.9 Gy (figure 3A), calculated with standard kidney mass. In 119 out of 323 Dutch patients the radiation dose to the kidneys was 19.9 ± 5.4 Gy (figure 3B) with standard kidney volume and

16.9 ± 5.2 Gy (figure 3C) with individual CT-based kidney volume.

**References**

1. Stabin MG, Siegel JA. Physical models and dose factors for use in internal dose assessment. Health Physics. 2003;85(3):294-310. doi:Doi 10.1097/00004032-200309000-00006.

2. Stabin MG, Sparks RB, Crowe E. OLINDA/EXM: the second-generation personal computer software for internal dose assessment in nuclear medicine. J Nucl Med. 2005;46(6):1023-7.

3. Bakker WH, Albert R, Bruns C, Breeman WA, Hofland LJ, Marbach P et al. [111In-DTPA-D-Phe1]-octreotide, a potential radiopharmaceutical for imaging of somatostatin receptor-positive tumors: synthesis, radiolabeling and in vitro validation. Life Sci. 1991;49(22):1583-91.

4. Bakker WH, Krenning EP, Reubi JC, Breeman WA, Setyono-Han B, de Jong M et al. In vivo application of [111In-DTPA-D-Phe1]-octreotide for detection of somatostatin receptor-positive tumors in rats. Life Sci. 1991;49(22):1593-601.

5. Konijnenberg MW, Bijster M, Krenning EP, De Jong M. A stylized computational model of the rat for organ dosimetry in support of preclinical evaluations of peptide receptor radionuclide therapy with (90)Y, (111)In, or (177)Lu. J Nucl Med. 2004;45(7):1260-9.

6. Sgouros G. Dosimetry of internal emitters. J Nucl Med. 2005;46 Suppl 1:18S-27S.

**
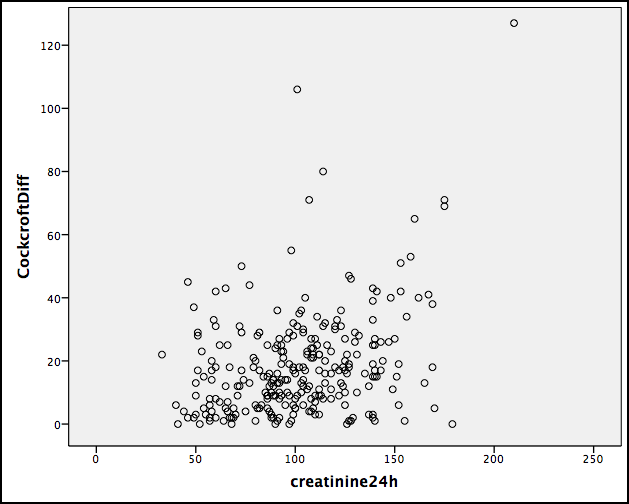

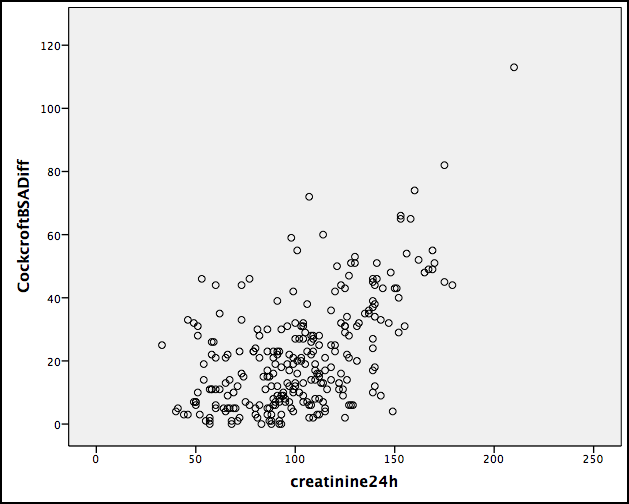

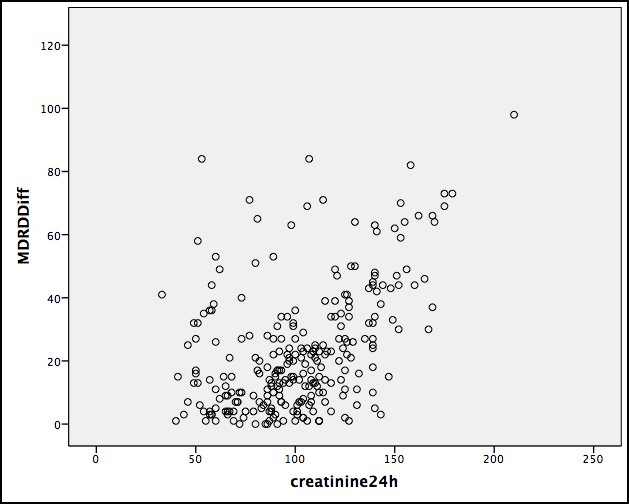

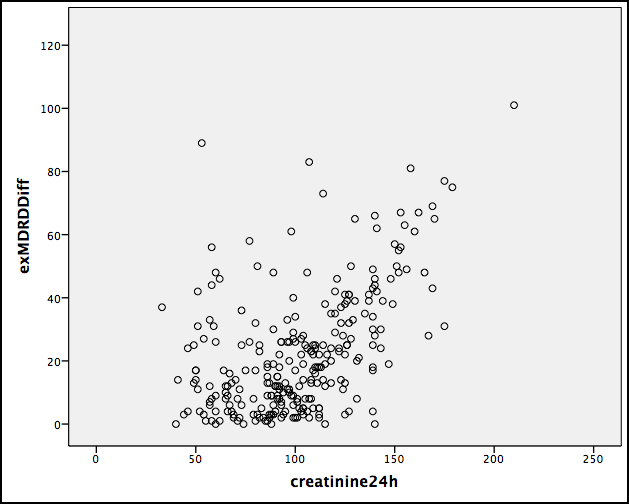
**

a

b

d

c

**Figure 1**

**
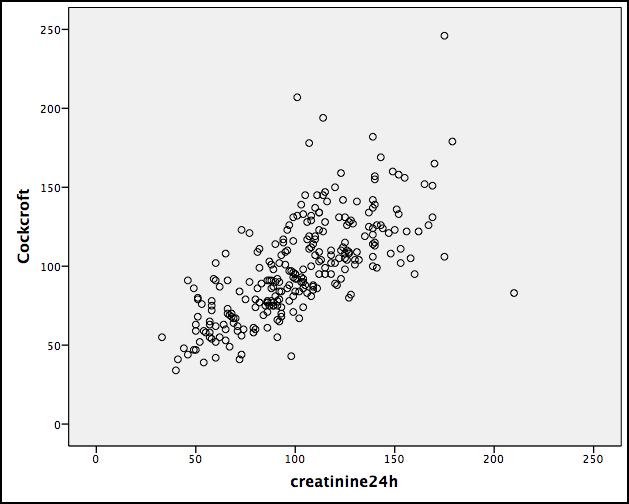

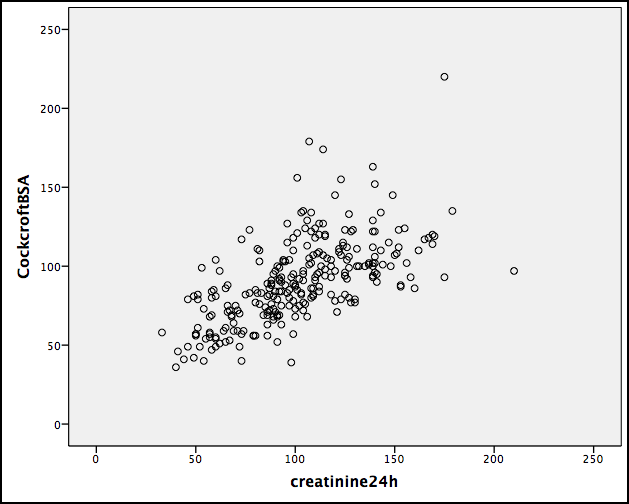

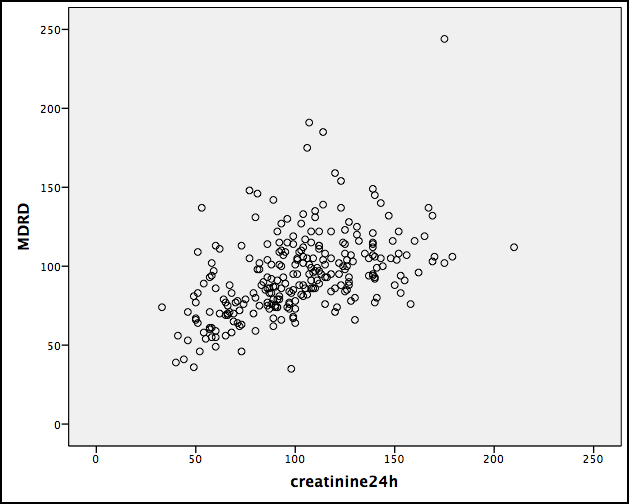

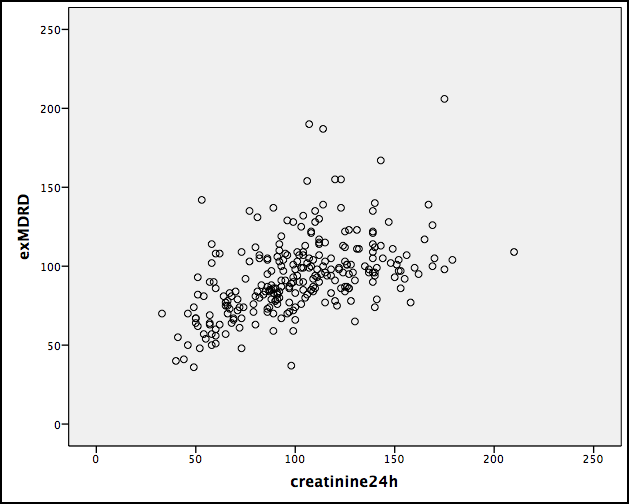
**

c

b

d

a

R_spearman_= 0.54

R_spearman_= 0.68

R_spearman_= 0.76

R_spearman_= 0.53

**Figure 2**

**
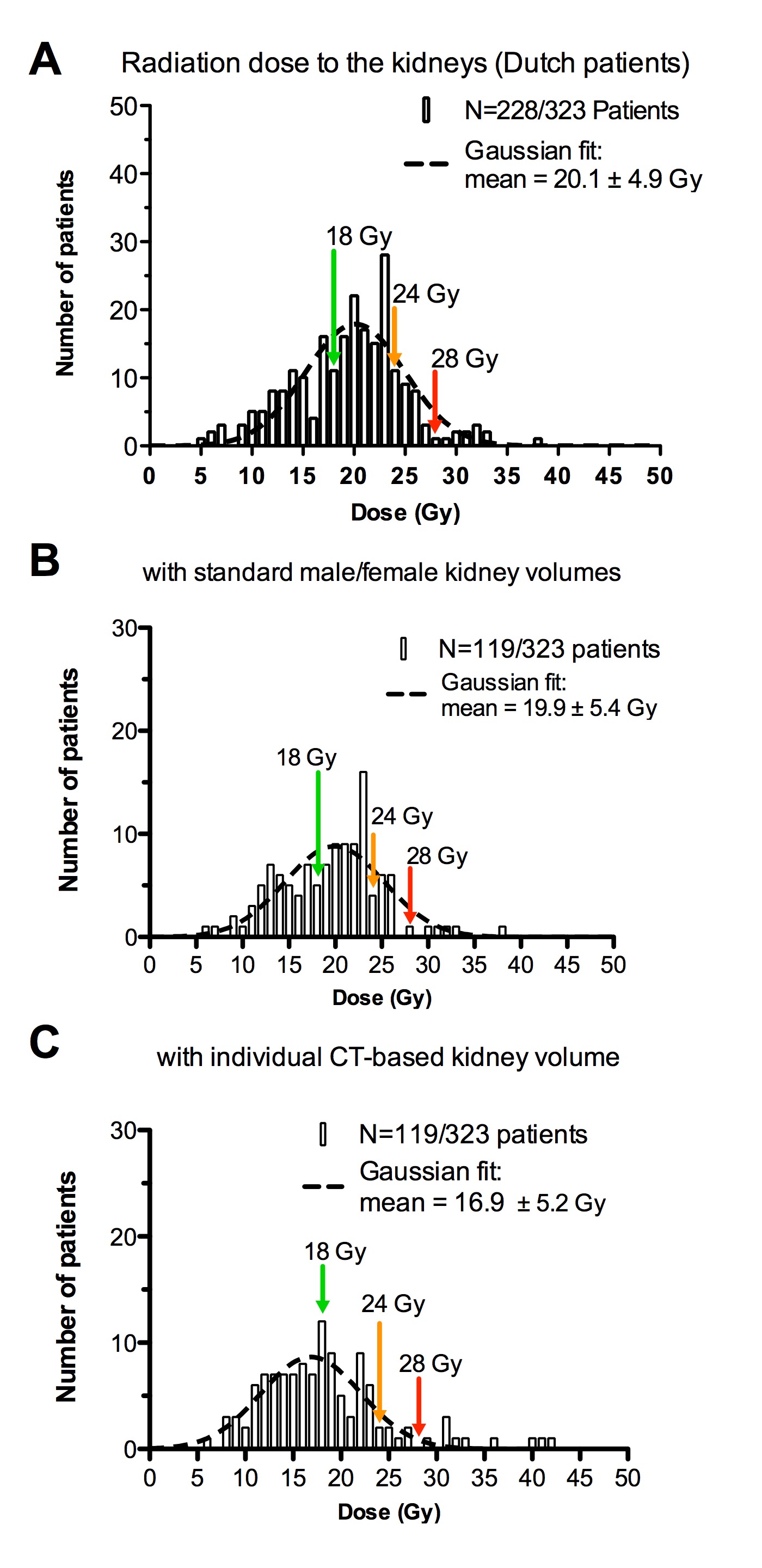
**

**Figure 3**

**Appendix A – Model comparison**

| Model | AICc | BIC | df |
| --- | --- | --- | --- |
| Linear | 18983.36 | 19012.45 | 5 |
| Polynomial | 18974.54 | 19015.76 | 7 |
| Spline | 18960.14 | 19018.28 | 10 |
| Nonlinear with plateau | 19606.72 | 19641.63 | 6 |
| Nonlinear without plateau | 19000.84 | 19029.94 | 5 |

| Subset Analysis: Model | AICc | BIC | df |
| --- | --- | --- | --- |
| Nonlinear without plateau | 15281.36 | 15337.47 | 10 |
| - including Kdose | 15281.93 | 15343.64 | 11 |
| -including BED | 15282.3 | 15344.01 | 11 |

**Appendix B – Final model**

Model output:

| Nonlinear mixed-effects model fit by REML | | | | | |
| --- | --- | --- | --- | --- | --- |
| Fixed effects | Coefficient | Std Err. | Df | t-value | p-value |
| Intercept | 107.745 | 4.665 | 2284 | 23.097 | 0.0000 |
| Age >70 vs. Age <=70 | -24.492 | 5.411 | 2284 | -4.526 | 0.0000 |
| HT (yes vs. no) | 0.932 | 4.466 | 2284 | 0.209 | 0.8346 |
| Diabetes (yes vs. no) | 3.299 | 3.933 | 2284 | 0.839 | 0.4017 |
| Baseline CTCAE | -46.190 | 6.552 | 2284 | -7.050 | 0.0000 |
| Newdosis > 600 vs. Newdosis <=600 | 0.868 | 4.582 | 2284 | 0.189 | 0.8498 |
| Decay rate | 0.001 | 0.000 | 2284 | 9.374 | 0.0000 |

Random effects:

Diagonal covariate matrix: estimated standard deviation of 26.17 and 0.001 for b0 and b1 respectively.

#Note; interactions tried between Time in weeks and Age, BasCTCAE, HT, Diabetes and NewDosis, do not improve model fit. Use of DMMed/HBA1c60 does not improve model fit over use of Diabetes.
